# Why Are We Still Implanting Permanent Synthetic Mesh for Hernia Repair Into Young Patients?

**DOI:** 10.3389/jaws.2026.16654

**Published:** 2026-04-14

**Authors:** Samuel G. Parker, Steve Halligan, Alastair C. J. Windsor, Sarah Zhao, Lawrence Nip, Marja A. Boermeester

**Affiliations:** 1 The Abdominal Wall Unit, Croydon University Hospital, London, United Kingdom; 2 Division of Medicine, Centre for Medical Imaging, The Rayne Institute, University College London, London, United Kingdom; 3 The Princess Grace Hospital, London, United Kingdom; 4 Department of Surgery, Amsterdam UMC, AGEM, University of Amsterdam, Amsterdam, Netherlands

**Keywords:** abdominal wall surgery, absorbable mesh, biosynthetic mesh, hernia, mesh

## Introduction

Inguinal, femoral, and abdominal hernias are significant health concerns, affecting millions annually worldwide [1]. Level 1 evidence suggests that using permanent synthetic mesh for surgical repair reduces recurrence significantly compared to not using mesh [2]. However, in this viewpoint we describe, (a) how permanent mesh can cause long-term complications and, (b) why absorbable mesh is preferable. The obvious (and some believe only) clinical indication for absorbable mesh is a contaminated surgical field but we believe this is not credible. We believe absorbable mesh has other indications, not least for younger patients. For clarity, we use the term “absorbable mesh” to cover meshes that to some extent act as tissue matrix, comprising of both long-acting biosynthetic meshes, such as Phasix™ (BD), Bio-A® (Gore), TIGR® (Novus Scientific), and Transorb™ (Medtronic), and next-generation reinforced biologic mesh; OviTex® (TELA Bio). These meshes exhibit critical differences. Long-acting biosynthetics promote tissue ingrowth, collagen deposition, and degrade over time via hydrolysis. Next generation biologic mesh is replaced by host collagen via tissue remodelling.

## The Permanent Mesh Problem

For over 2 decades, we have known that permanent plastic meshes trigger a foreign body reaction and inflammatory response. In the short-term, this probably exacerbates pain, particularly after inguinal hernia repair. Longer-term complications (including contraction, encapsulation, bowel obstruction, erosion, infection, sinus, seroma, migration, and chronic pain), occur in around 6% of patients following ventral hernia repair [3]. These complications are exacerbated by an inflammatory capsule that surrounds the mesh and, occasionally, a biofilm that encourages bacteria, encouraging chronic infection [4]. Complications become increasingly prevalent with age, occurring many years later [3]. Complications cause considerable morbidity, with mesh infection being particularly problematic, and explantation entails major surgery and can destroy the anterior abdominal wall completely.

Operating through permanent mesh is not trivial. Mesh adheres to bowel, risking inadvertent enterotomy and contamination [5]. Minimal access surgery is frequently impossible, necessitating open procedures and increasing morbidity [6]. Furthermore, incisions through implanted mesh and adjacent fibrosis heal poorly, increasing wound morbidity and hernia recurrence [6]. We must also consider the significant chance of future non-hernia-related surgery, especially in younger patients. In the western world lifetime cancer risk is 50%, frequently requiring abdominal surgery both for cure and recurrence [7]. Copious examples for non-malignant abdominal surgery also exist and include trauma, appendicitis, diverticulitis, gastric ulcer perforation, inflammatory bowel disease, and organ transplantation. There are endless examples, few of which are seldom; for example, the lifetime risk of acute appendicitis is 7%–9% [8]. Moreover, future surgery in a contaminated environment, or on immunosuppressed patients, risks infecting an existing mesh [9].

Plastic meshes are not biocompatible and generate an inflammatory capsule and scar plate [10]. Estimating the extent to which this hinders function is challenging as there is little consensus on how to measure abdominal wall function and quality of life after hernia repair [11]. Nevertheless, patients describe abdominal wall stiffness, mesh awareness, and chronic discomfort following permanent synthetic mesh. Bio-incompatibility also causes poor physiological adaptation to weight gain or loss, growth, or pregnancy. Tearing at the mesh-tissue interface can occur, creating marginal defects and triggering hernia recurrence. Being mindful that re-operation rates after ventral hernia repair approach 20% at 15 years [12], permanent mesh obliterates abdominal wall planes due to surrounding inflammation, meaning that these planes are excluded from future use during recurrent hernia repair and the surgical challenge will be significantly increased.

## Absorbable Mesh

Absorbable mesh exhibits a range of potential advantages over permanent synthetic mesh. As no or minimal foreign material remains *in vivo* long-term, patients are less exposed to the complications associated with permanent implants, such as mesh shrinkage, migration, erosion into surrounding viscera, and recurrence driven by chronic shear forces at the mesh-abdominal wall interface [13, 14]. It is therefore plausible that the reduced chronic inflammatory response and lower visceral adhesion burden associated with absorbable meshes may lessen the risk of nerve impingement, neuritis, and chronic pain [14]. Furthermore, mesh degradation and reduced long-term inflammation means that tissue planes are preserved for future repair, facilitating surgical options and reducing the complexity associated with re-operation through permanent mesh, as described in the paragraphs above. This attribute is especially relevant for parastomal hernia, which occur and recur frequently. While guidance suggests using permanent synthetic mesh for parastomal hernia prophylaxis [15], erosion into bowel is possible given such close proximity. Erosion is less likely with absorbable mesh, diminishing the likelihood of peristomal complications (infection, fistula, pain). These meshes may also be best for prophylaxis of incisional hernia after midline laparotomy (a hot topic for abdominal wall surgeons). The mesh remains *in situ* during wound healing, reinforcing the midline with new tissue ingrowth, but the mesh will subsequently be degraded or will be remodelled, before the need for further abdominal surgery.

## Discussion

General surgeons have been slow to adopt absorbable mesh for clean operating fields. Reluctance is mainly due to cost since these meshes are significantly more expensive than permanent synthetic. However, increased adoption may lower cost due to economies of scale; furthermore, the net cost may decrease if the morbidity of future surgery is reduced. Surgeons are also concerned that absorbable mesh may increase recurrence as, conceptually, they believe long-term abdominal wall reinforcement disappears along with the mesh. Despite absorbable mesh being used in clinical practice for over a decade, little is known regarding long term performance versus permanent alternatives, with very few publications reporting long-term follow-up. Lastly, a conservative attitude may exist against exploring absorbable mesh beyond its use in contaminated fields, as surgeons practice what they know or have been taught.

At the European Hernia Society (EHS) annual conference in Paris this year, sessions exploring patient reported outcomes (PROMs) and abdominal wall biomechanics were over-subscribed, indicating that surgeons are intensely interested in these topics, understanding that long-term functional outcomes are increasingly relevant to modern day surgical practice. It is our view that absorbable mesh may well improve long-term patient focused outcomes such as quality-of-life scores, abdominal wall function, and chronic pain/discomfort. Moreover, patients are now advocating for these meshes. As lawsuits against permanent mesh implantation rise, patients are increasingly aware of potential harms and are demanding alternatives. This may be particularly relevant to young patients, the immunosuppressed and to those with conditions such as Crohn’s disease. One patient advocate writes, “It is important that patients are confident in the knowledge that what is implanted into their bodies is not going to cause symptoms from inflammation, and foreign body rejection.”^1^

So far, the long-term safety profile for these new meshes has been encouraging, increasing its adoption substantially [16]. Due to the concerns we have raised, we have found that use of absorbable mesh has increased. As abdominal wall reconstruction continues to mature into a distinct sub-specialty, we must collect data that compares absorbable and permanent synthetic mesh directly. The hernia community needs prospective, generalisable, large-scale data with follow-up sufficient to understand outcomes after primary or incisional hernia repair, especially in younger patients. Ultimately, this viewpoint has been formed from our own clinical experience and a growing body of published literature [16, 17].

## Footnotes

1 Jackie Bullock: chronic pain patient and founder of the European Hernia Society’s hernia patient support group on Facebook.
